# Novel Agents Targeting the IGF-1R/PI3K Pathway Impair Cell Proliferation and Survival in Subsets of Medulloblastoma and Neuroblastoma

**DOI:** 10.1371/journal.pone.0047109

**Published:** 2012-10-08

**Authors:** Anna Wojtalla, Fabiana Salm, Ditte G. Christiansen, Tiziana Cremona, Paulina Cwiek, Tarek Shalaby, Nicole Gross, Michael A. Grotzer, Alexandre Arcaro

**Affiliations:** 1 Division of Pediatric Hematology/Oncology, Department of Clinical Research, University of Bern, Bern, Switzerland; 2 Department of Oncology, University Children's Hospital Zurich, Zurich, Switzerland; 3 Department of Pediatrics, Pediatric Oncology Research, University Hospital CHUV, Lausanne, Switzerland; University of Navarra, Spain

## Abstract

The receptor tyrosine kinase (RTK)/phosphoinositide 3-kinase (PI3K) pathway is fundamental for cancer cell proliferation and is known to be frequently altered and activated in neoplasia, including embryonal tumors. Based on the high frequency of alterations, targeting components of the PI3K signaling pathway is considered to be a promising therapeutic approach for cancer treatment. Here, we have investigated the potential of targeting the axis of the insulin-like growth factor-1 receptor (IGF-1R) and PI3K signaling in two common cancers of childhood: neuroblastoma, the most common extracranial tumor in children and medulloblastoma, the most frequent malignant childhood brain tumor. By treating neuroblastoma and medulloblastoma cells with R1507, a specific humanized monoclonal antibody against the IGF-1R, we could observe cell line-specific responses and in some cases a strong decrease in cell proliferation. In contrast, targeting the PI3K p110α with the specific inhibitor PIK75 resulted in broad anti-proliferative effects in a panel of neuro- and medulloblastoma cell lines. Additionally, sensitization to commonly used chemotherapeutic agents occurred in neuroblastoma cells upon treatment with R1507 or PIK75. Furthermore, by studying the expression and phosphorylation state of IGF-1R/PI3K downstream signaling targets we found down-regulated signaling pathway activation. In addition, apoptosis occurred in embryonal tumor cells after treatment with PIK75 or R1507. Together, our studies demonstrate the potential of targeting the IGF-1R/PI3K signaling axis in embryonal tumors. Hopefully, this knowledge will contribute to the development of urgently required new targeted therapies for embryonal tumors.

## Introduction

Second to accidents, cancer is still the leading cause of death for children. Embryonal tumors represent approximately 30% of childhood malignancies and often display resistance to current therapeutic regimens. Therefore, embryonal tumors are associated with lower survival rates compared to other childhood cancers. Treatment failure for disseminated disease is frequent, and results in survival rates <20%. Thus, novel therapeutic options are urgently needed for this group of tumors to improve survival rates and quality of life of patients. Embryonal tumors are dysontogenetic tumors whose pathological features resemble those of the developing organ or tissue of origin and include the entities medulloblastoma and neuroblastoma. Medulloblastoma is the most common malignant brain tumor in children and accounts for approximately 20% to 25% of all pediatric central nervous system tumors. Neuroblastoma is an embryonal tumor that originates from developing neural crest tissues. It is the most common extracranial solid tumor and is responsible for 15% of all cancer-related deaths in childhood. The fact that these cancers occur in infants and young children suggests that only a limited number of genetic changes may lead to tumor development, making these cancers an attractive model to identify new molecular targets. The development of novel targeted therapies is of particular importance for embryonal tumors, as these malignancies are orphan diseases. Common intracellular signaling pathways and chromosomal deletions including 1p36 and 11q loss have been previously identified in different embryonal tumors, including medulloblastoma and neuroblastoma [1]–[10].

**Figure 1:**
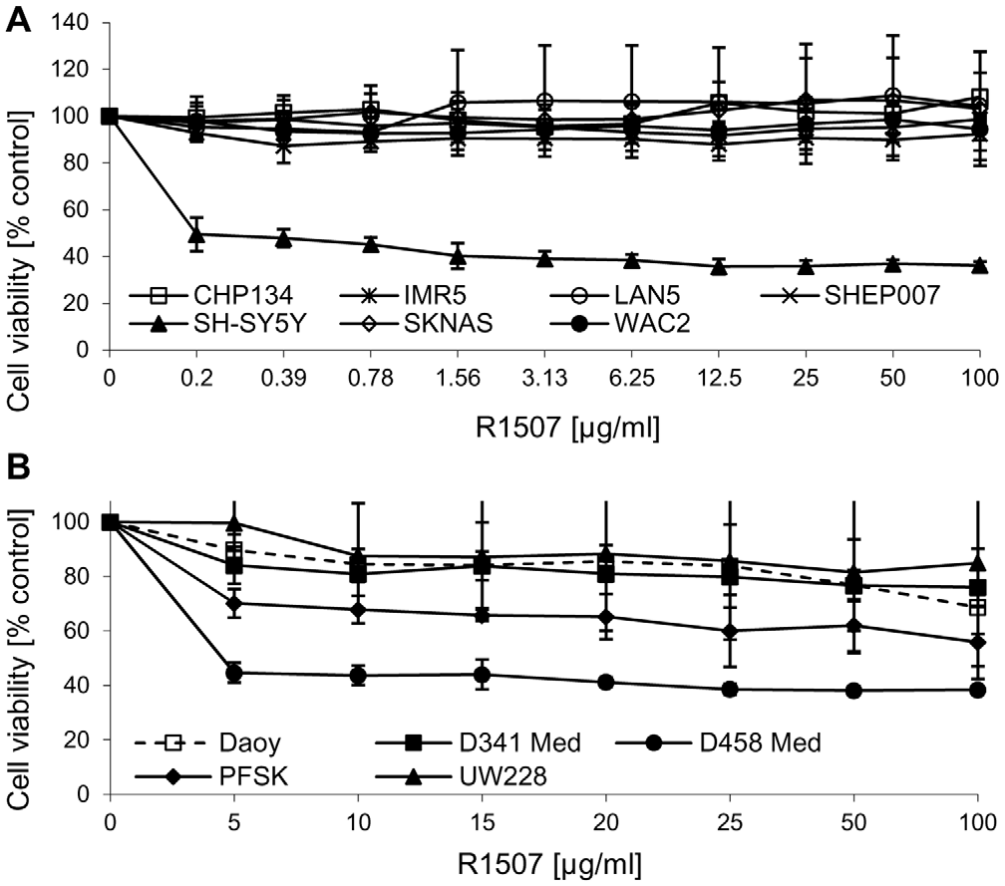
The effect of R1507 on cell proliferation of NB and MB cells. A panel of NB cell lines (A) and MB cell lines (B) were incubated with increasing concentrations of the antibody R1507 inhibiting the IGF-1R in serum-containing medium. Cell viability was assessed using the MTS assay after 2 days. The data represent the mean with SD from at least 6 replicates and 3 independent experiments.

Several intracellular signaling pathways have indeed been demonstrated to play a key role in embryonal tumor biology. Indeed, polypeptide growth factors such as insulin-like growth factor-1 (IGF-1), epidermal growth factor (EGF), platelet-derived growth factor (PDGF), neuregulins and neurotrophins have been shown to control embryonal tumor proliferation, survival, differentiation and metastasis [11]–[15] by binding to specific receptor tyrosine kinases (RTKs). Moreover, expression of the ErbB-2 and ErbB-4 RTKs in embryonal tumor samples was shown to correlate with reduced patient survival, while Trk receptor expression correlated with a less aggressive tumor phenotype [13]. Therefore a better understanding of the involvement of RTKs and their downstream targets in human embryonal tumor biology may yield important clues for the development of new drugs for the disease. Targeting receptor tyrosine kinases such as the IGF-1R is a promising approach to develop novel anti-cancer therapies in embryonal tumors, such as neuroblastoma and sarcoma [15]–[23]. Indeed the first results from clinical trials evaluating the safety and efficacy of IGF-1R neutralizing antibodies in children and adolescents with embryonal tumors have been reported [24], [25]. In these trials, the humanized IGF-1R neutralizing antibody R1507 displayed minimal toxicities and some responses in ESFT were observed [24], [25]. Importantly, no dose-limiting toxicities were identified and the maximum tolerated dose was not reached [24]. Human embryonal tumor cells have been reported to express a variety of growth factor receptors, some of which can be activated by mutations, over-expression and/or establishment of autocrine loops [13]. Amongst these polypeptide growth factor receptors are the RTKs IGF-1R, EGFR, ALK, ErbB-2, ErbB-4, c-Kit, PDGFR, Trk and fibroblast growth factor receptor (FGFR) [26]–[41]. Therefore, given that embryonal tumor cells express a variety of different growth factor receptors, targeting individual receptors may not provide a successful therapeutic strategy in all embryonal tumor entities. A potentially complementary approach would be to identify signaling molecules which lie downstream of several different growth factor receptors and which are essential for transmitting their proliferative and/or survival message. Combinatorial targeting of receptor tyrosine kinases (such as the IGF-1R) and their downstream signaling mediators is a very promising approach to develop more efficient anti-cancer therapies [16], [17], [22], [42]–[44].

**Figure 2:**
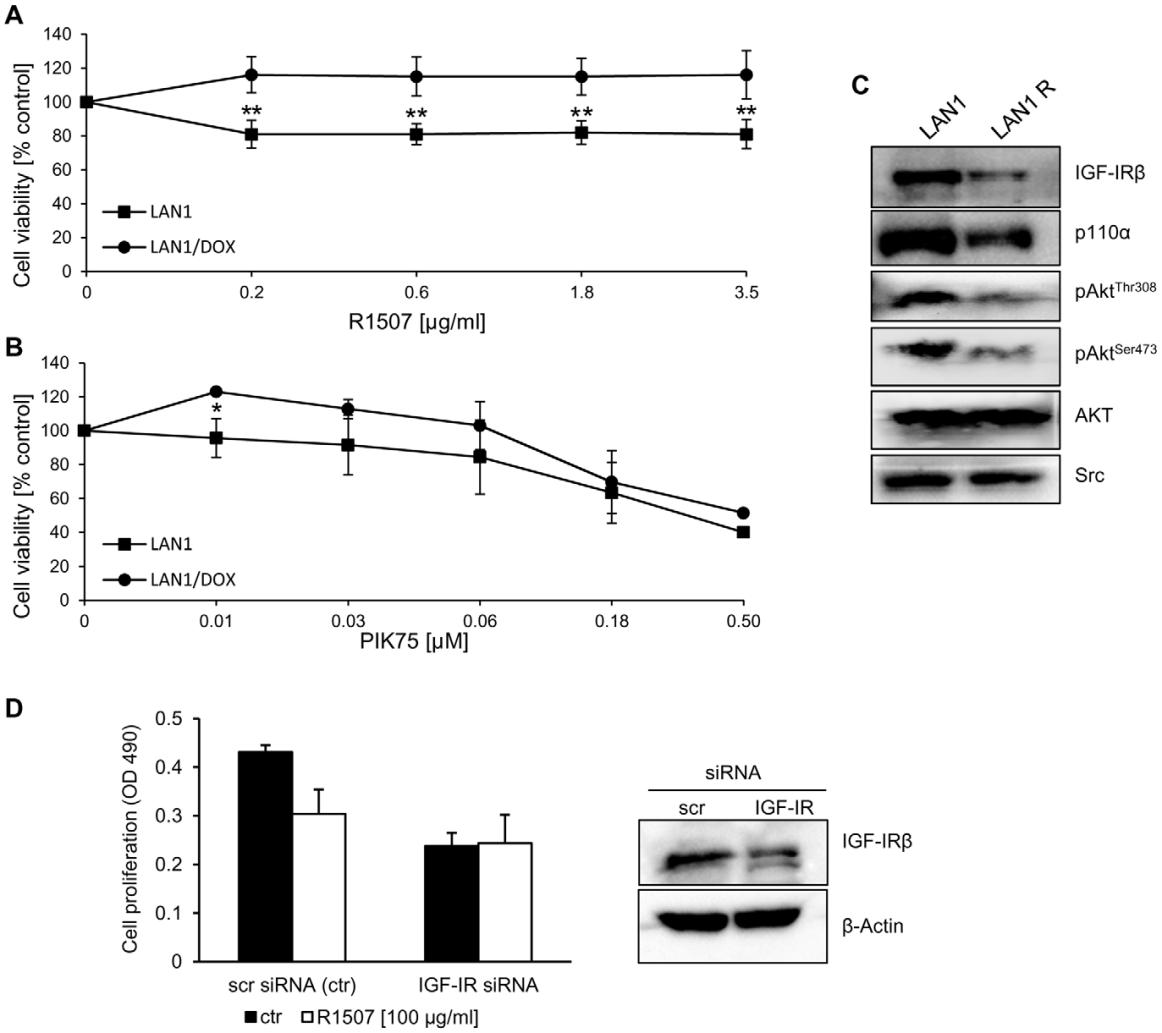
Sensitivity to R1507 and PIK75, and presence of IGFR in neuroblastoma cell lines LAN1 and LAN1R, a LAN1 cell line resistant to doxorubicin. (A) R1507 treatment for 48 hours. (B) PIK75 treatment for 48 hours. Error bars represent ±S.D. of means from 3 experiments, each with 3 replicates, except that there was only one experiment with 500 nM PIK75 in B. (C) Western blot analysis of components of the IGF-1R/PI3K pathway in LAN1 and LAN1R whole cell extracts. Src was used as internal loading control. (D) Transfection of LAN1 cells with siRNA targeting the IGF-1R (non-targeting siRNA was used as control. Expression levels of the IGF-1R were assessed by Western blot analysis in LAN1 whole cell lysates after 96 h). Cell proliferation in LAN1 cells upon IGF-1R silencing was assessed in absence or presence of R1507 after 96h by MTS. (*p<0.05).

The phosphoinositide 3-kinase (PI3K) plays a crucial role in controlling cell proliferation, survival and motility/metastasis downstream of many different growth factor receptors and oncogenic Ras mutants [45]–[48]. PI3K signaling activates a crucial intracellular signaling pathway involving phosphoinositide-dependent protein kinase-1 (PDK1), Akt, the mammalian target of rapamycin (mTOR) and the ribosomal protein S6 kinase (S6K), which controls cell growth, proliferation and survival [45]–[47]. The importance of PI3K/Akt/mTOR signaling in cancer is highlighted by the fact that mutations in the tumor suppressor gene *PTEN* occur frequently in human tumors, including glioblastoma [45], [49]–[51]. PTEN is a phosphatase that antagonizes the action of PI3K by de-phosphorylating the D-3 position of poly-phosphoinositides [45], [49], [50]. Reduced expression of PTEN resulting in activation of PI3K signaling was recently described in embryonal tumors such as medulloblastoma and neuroblastoma [52], [53]. Moreover, various reports have described activating mutations in the *PIK3CA* gene encoding the catalytic p110α isoform of PI3K in a variety of human cancers, including, breast, colon and ovarian cancer, as well as embryonal tumors [51], [54], [55]. In addition, PI3K/Akt/mTOR signaling has been demonstrated to mediate the proliferation of embryonal tumor cells [56], [57] and to contribute to signaling by ErbB-2 and IGF-1R [58]–[60]. Activation of Akt was also reported in embryonal tumors, correlating with poor outcome in some entities [61]. Thus, targeting the PI3K/Akt/mTOR signaling pathway may represent an attractive novel approach to develop novel therapies for embryonal tumors [62]. Indeed, there now exist multiple pharmacological inhibitors of the PI3K/Akt/mTOR pathway which have entered clinical trials for adult and pediatric cancer [44], [46], [48], [63]–[65]. The PI3K/Akt/mTOR pathway is also an important contributor to the resistance of human tumors to drugs targeting receptor tyrosine kinases [66]–[68]. Inhibitors of the PI3K/Akt/mTOR signaling pathway have also been shown to be effective in combination with IGF-1R inhibitors [20], [43].

**Figure 3:**
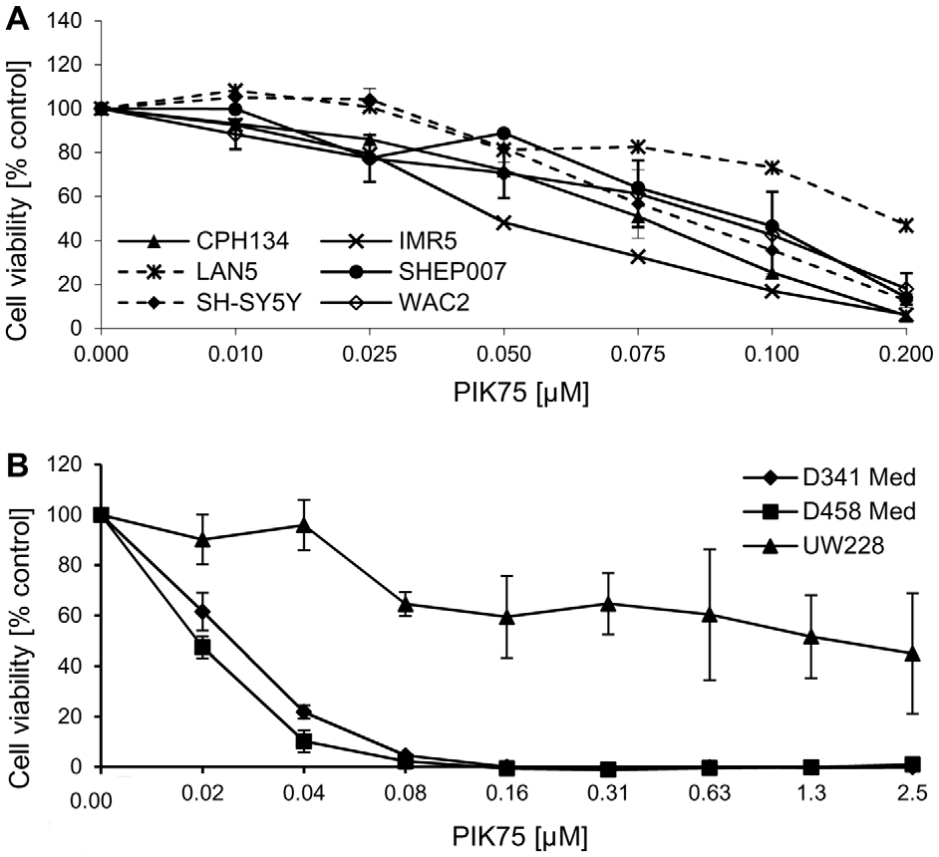
Cell proliferation of NB and MB cells after inhibition of the PI3K p110α. A panel of NB cell lines (A) and MB cell lines (B) were incubated with increasing concentrations of the specific pharmacological PI3K p110α inhibitor PIK75 in serum-containing medium. Cell viability was assessed using the MTS assay after 2 (NB) or 3 (MB) days. The data represent the mean with SD from at least four replicates and 1–3 independent experiment.

In the present report, we have evaluated the anti-proliferative potential of the humanized anti-IGF-1R antibody R1507 and of PIK75, a class I_A_ PI3K inhibitor, in medulloblastoma and neuroblastoma cell lines. We present evidence that these agents are effective as monotherapies in subsets of embryonal tumor cell lines and can be effectively combined with standard chemotherapeutic drugs.

**Figure 4:**
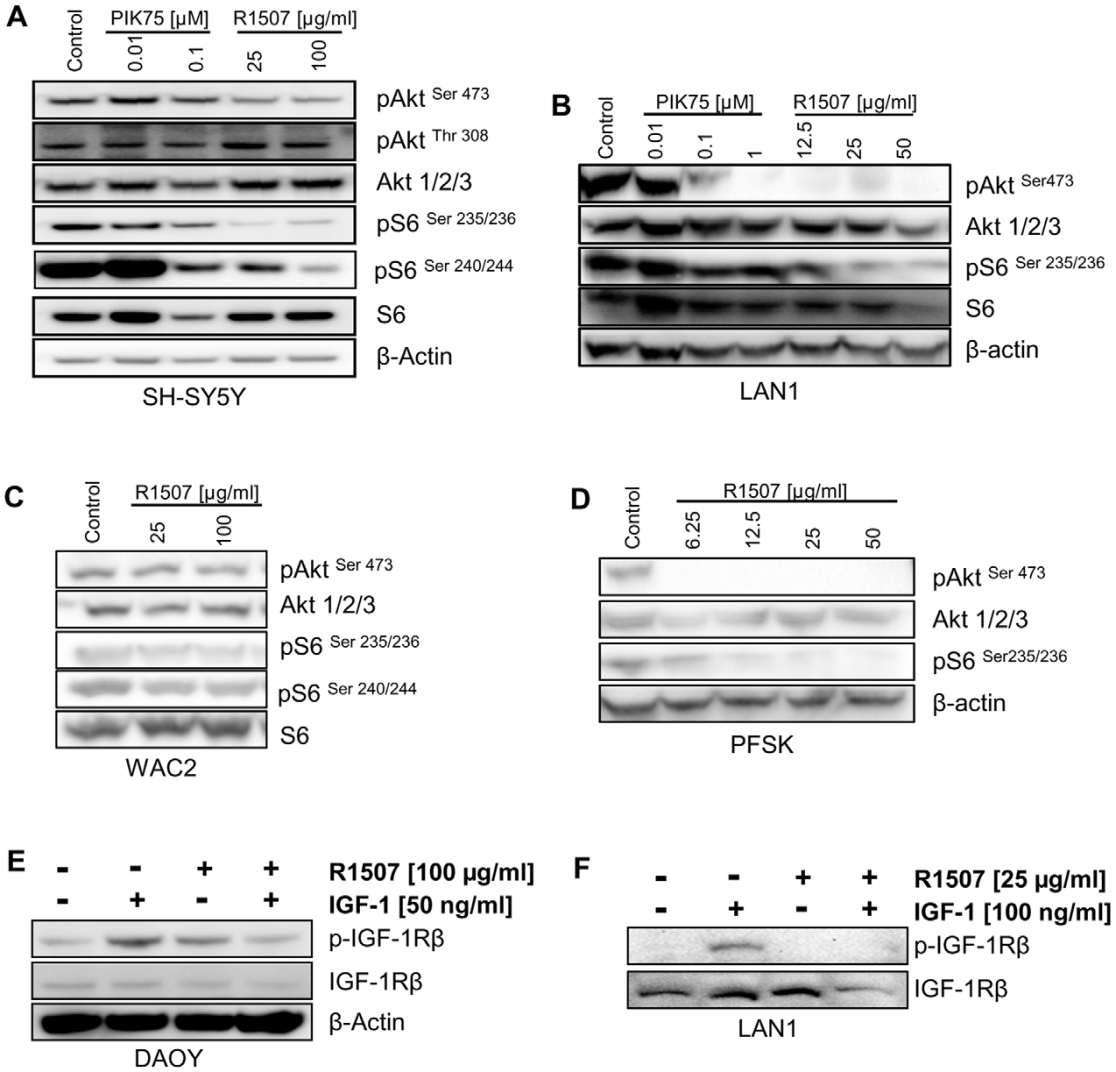
PI3K and IGF-1R inhibition impair receptor activation and downstream signaling. The NB cells SH-SY5Y (A), LAN1 (B); WAC2 (C) and the MB cells PFSK (D) grown in serum-containing medium were incubated with increasing concentrations of the PI3K p110α inhibitor PIK75 and the IGF-1R antibody R1507. After 24 hours the cells were harvested and whole cell lysates analysed by SDS-PAGE and Western blotting for the proteins indicated. Serum-starved DAOY (E) or LAN1 (F) cells were pre-treated with vehicle or R1507 at the concentrations indicated for 1h and stimulated with IGF-1 (50 ng/ml or 100 ng/ml) for 10 min at 37°C. Cell lysates were analysed by SDS-PAGE and Western Blot for phosphorylated IGF-1R beta and total receptor.

## Materials and Methods

### Antibodies and reagents

Antibodies specific for IGF1Rβ, PI3K p110α, Akt1/2/3, ERK 1/2, S6 protein, Caspase-3 (Santa Cruz Biotechnology, CA, USA), phospho-ERK1/2 (Thr^202^/Tyr^204^), phospho-Akt (Ser^473^; Thr^308^), poly (ADP-ribose) polymerase (PARP), S6 protein, phospho-S6 (Ser^235^/Ser^236^; Ser^240^/Ser^244^) p-IGF-1R(Cell Signaling Technology), β-actin (Sigma Aldrich).

**Figure 5:**
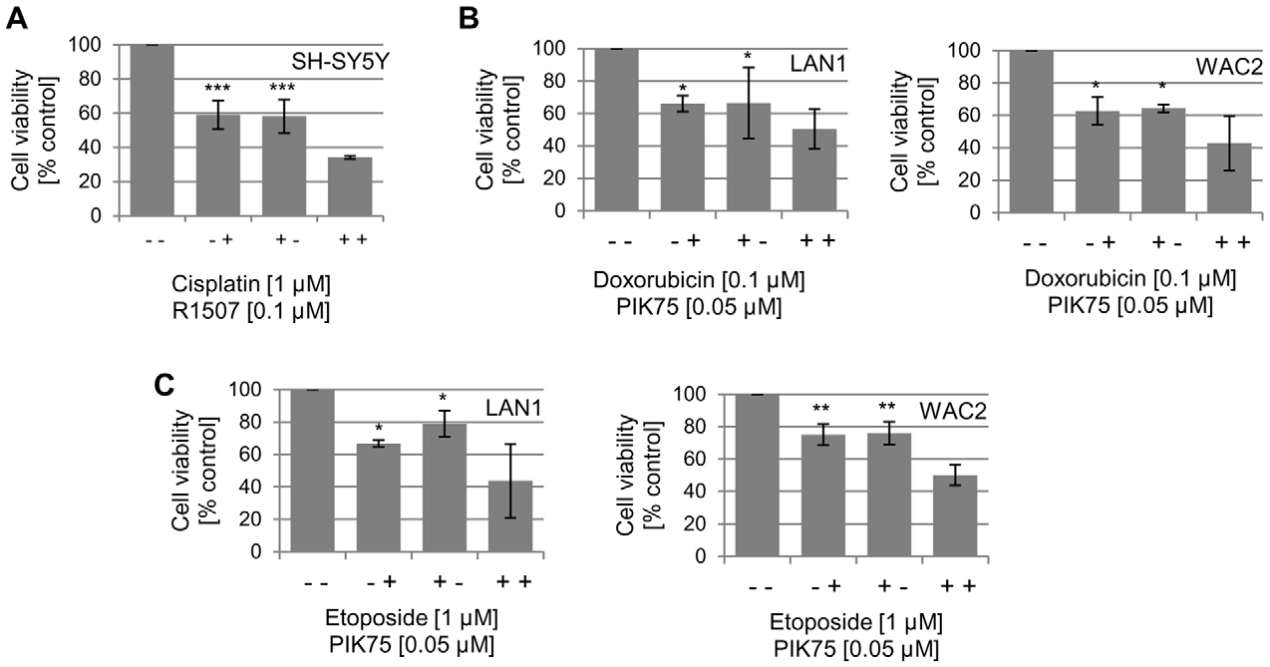
Treatment of NB cells with R1507 or PIK75 in presence of chemotherapy results in additive effects. NB cells grown in serum-containing medium were incubated with the IGF-1R antibody R1507 (0.1 μg/ml) (A) or the PI3K inhibitor PIK75 (0.05 μM) (B+C) in presence or absence of cisplatin (1 μM), etoposide (1 μM), or doxorubicin (0.1 μM). Cell proliferation was assessed using the MTS assay after 48 h. The data represent the mean of 8 replicates with SD from 3 independent experiments. (*p<0.05).

**Figure 6:**
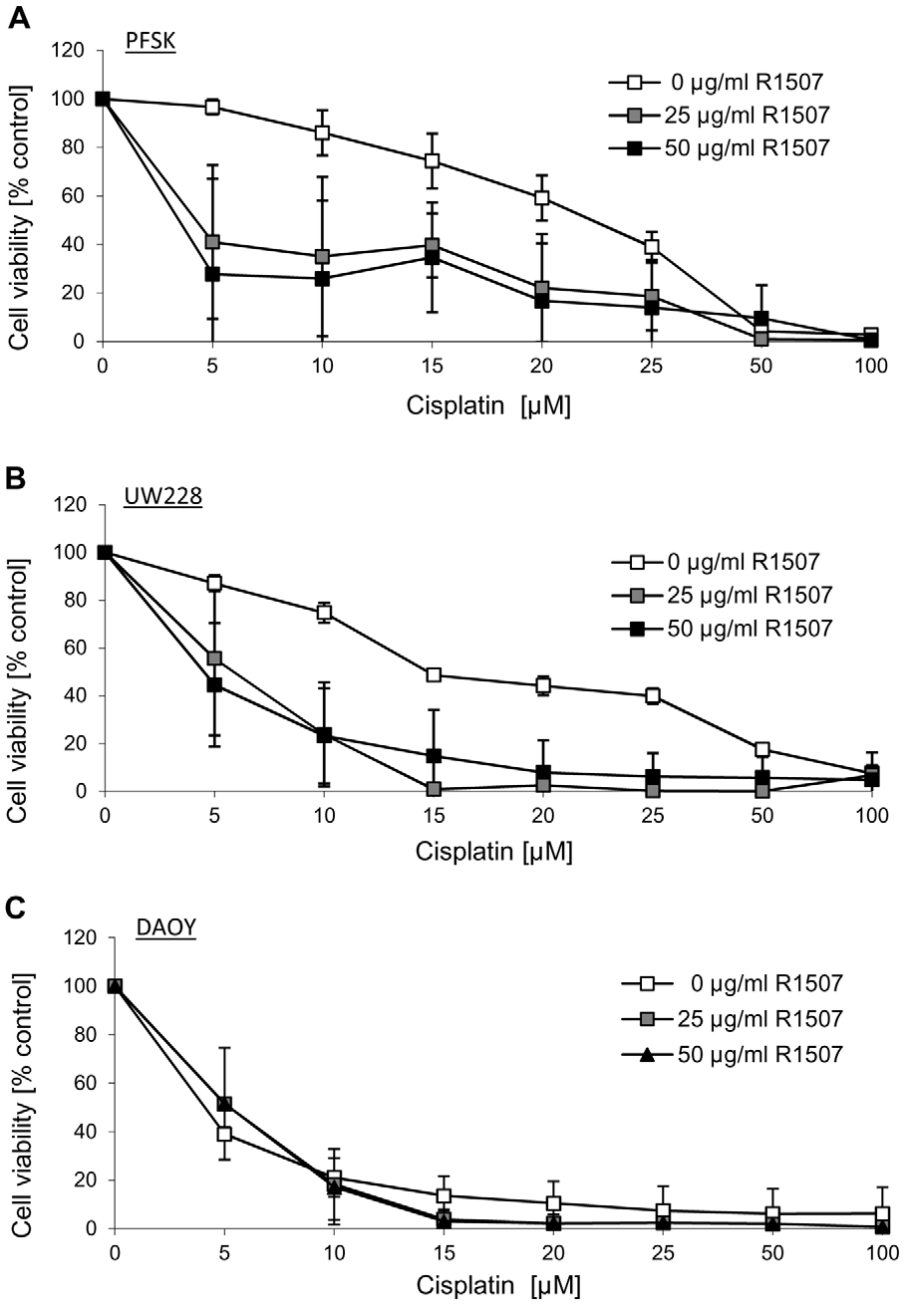
Additive/sensitization effects after combinatorial treatment with R1507 and chemotherapy in MB cells. The MB cell lines PFSK (A), UW228 (B) and DAOY (C) grown in serum-containing medium were incubated with increasing concentrations of cisplatin in presence or absence of the IGF-1R antibody R1507 (25 µg/ml or 50 µg/ml). Cell proliferation was assessed using the MTS assay after 48 h. The data represent the mean of 6 replicates with SD from 3 independent experiments.

The PI3K inhibitor PIK-75 [69] was dissolved in DMSO (Sigma, Buchs, Switzerland) at 10 mM and diluted into cell culture medium just before use. R1507, a fully human IgG1 monoclonal antibody to IGF-1R, was obtained from Roche, and was diluted directly into the medium immediately before use. The chemotherapeutic agents cisplatin (Bristol-Myers Squibb), doxorubicin (Pfizer) and etoposide (Calbiochem) were used in combination with PIK75 or R1507 at the indicated concentrations.

**Figure 7:**
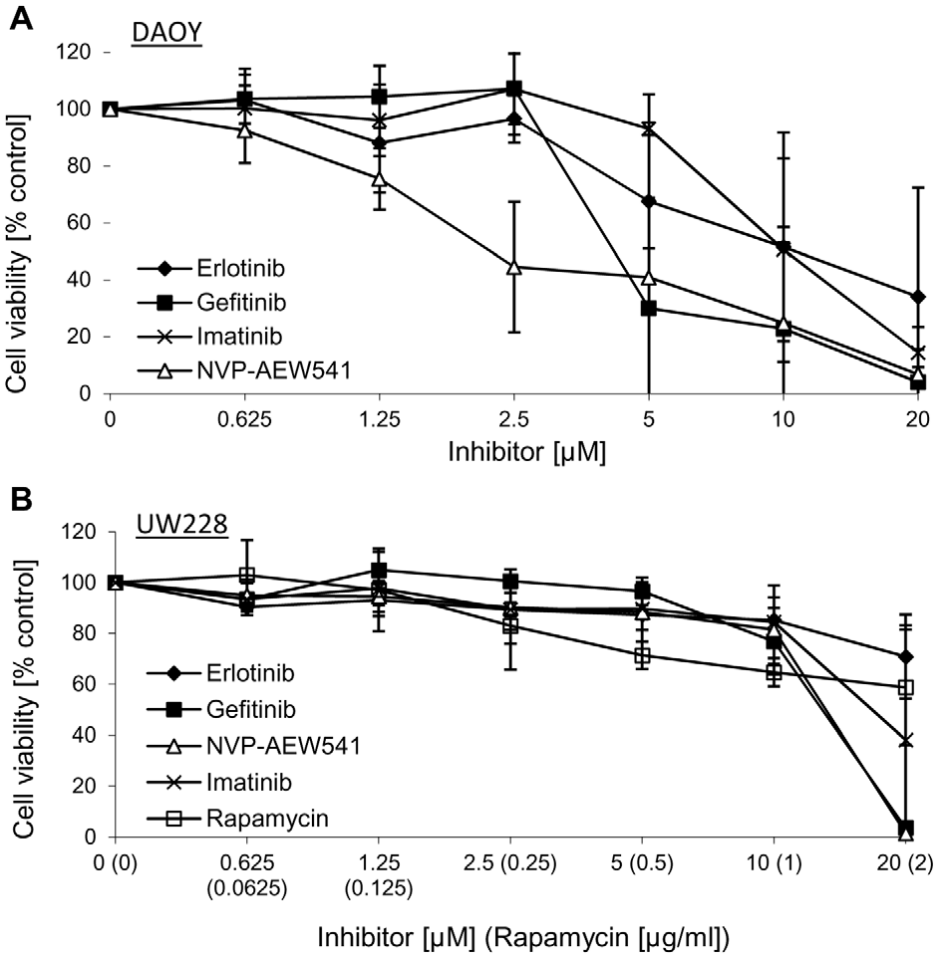
Apoptosis upon PI3K inhibition. (A+B) The NB cell lines LAN1 (A, left panel; B, left panel) and SH-SY5Y (B, right panel) as well as the MB cell line PFSK (A, right panel) grown in serum-containing medium were incubated with increasing concentrations of the PI3K p110α inhibitor PIK75 and the IGF-1R antibody R1507, or cisplatin. After 24 hours the cells were harvested and whole cell lysates analysed by SDS-PAGE and Western blotting for the proteins indicated. (C) The NB cell line WAC2 grown in serum-containing medium was incubated with increasing concentrations of the PI3K p110α inhibitor PIK75. Caspase 3/7 activity was assessed using the Caspase 3/7 Glo assay after 48 h.

**Figure 8:**
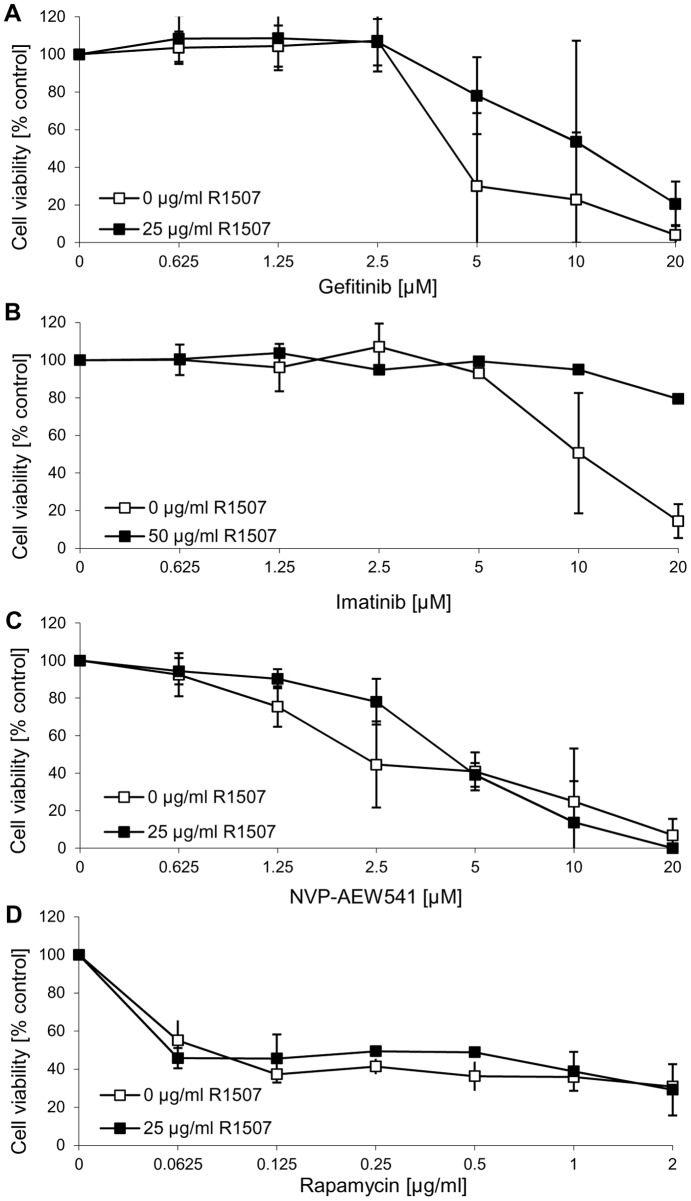
Targeted therapies in medulloblastoma. The MB cell lines Daoy (A) and UW228 (B) were incubated with increasing concentrations of the EGFR inhibitors gefinitib or erlotinib, the Abl inhibitor imatinib, the IGF-1R inhibitor NVP-AEW541 and the mTOR inhibitor rapamycin. Cell proliferation was assessed using the MTS assay after 72 h. The data represent the mean of 6 replicates with SD from 3 independent experiments.

### Cell lines, cell culture, cell proliferation and apoptosis

Human neuroblastoma cells were from the American Type Culture Collection and were kindly provided by Dr. Brodeur, Children's Hospital of Philadelphia. The cells were grown in RPMI (Life Technologies/Invitrogen) supplemented with 10% (v/v) fetal calf serum (FCS) and penicillin/streptomycin/L-glutamine and passaged every 3–5 days by trypsinization [20], [70].

**Figure 9:**
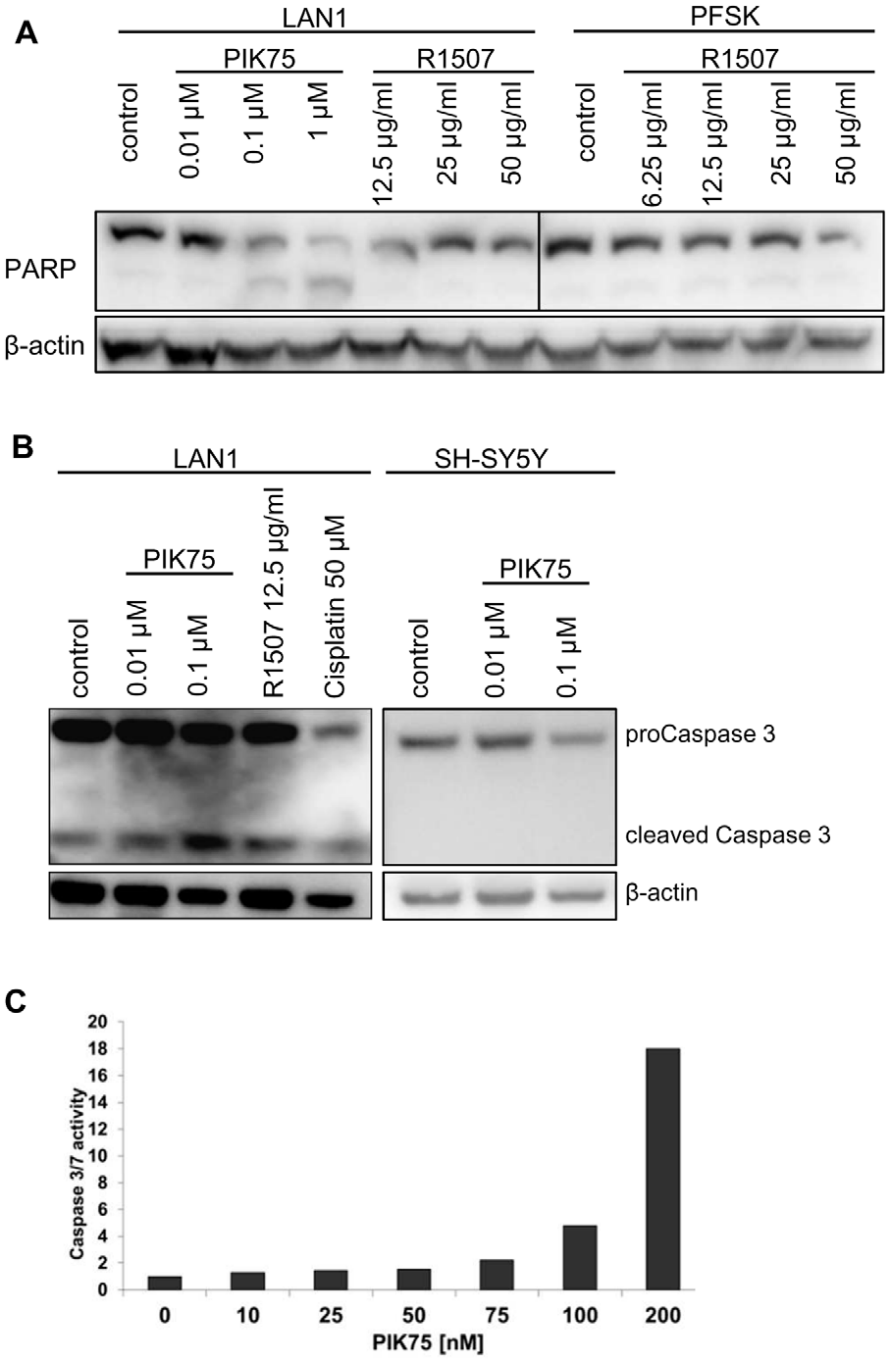
Effects of R1507 in combination with targeted therapies in medulloblastoma. The R1507-insensitive cell line DAOY was incubated with increasing concentrations of the EGFR inhibitor gefinitib (A), the Abl inhibitor imatinib (B), the IGF-1R inhibitor NVP-AEW541 (C) and the mTOR inhibitor rapamycin (D) in presence or absence of the IGF-1R antibody R1507. Cell proliferation was assessed using the MTS assay after 72 h. The data represent the mean of 6 replicates with SD from 3 independent experiments.

The medulloblastoma cell lines' provenience has been previously described. DAOY, UW-228 and PFSK human cell lines were purchased from the American Type Culture Collection. D341 Med and D458 Med medulloblastoma cells were the kind gift of Dr. Henry Friedman (Duke University, Durham, NC) [71], [72]. Cell lines that were not purchased from the American Type Culture Collection in 2009 were tested for their authentication by karyotypic analysis using molecular cytogenetic techniques, such as comparative genomic hybridization. DAOY medulloblastoma cell line was grown in Richter's MEM Zinc option medium (Invitrogen) with 10% FCS (fetal bovine serum; Sigma) and penicillin/streptomycin (Invitrogen). PFSK primitive neuroectodermal tumor (PNET) cell line [73] was grown in RPMI 1640 (Invitrogen) with 10% FCS and penicillin/streptomycin/L-glutamine. The UW-228 medulloblastoma cell line was grown in DMEM (Dulbecco's modified Eagle's medium; Invitrogen) with 10% FCS and penicillin/streptomycin/L-glutamine. D341 Med and D458 Med medulloblastoma cell lines were grown like DAOY but with the addition of 100M non-essential amino acids (GIPCO^TM^ MEM Invitrogen). All cells were grown in a humidified atmosphere at 37° and 5% CO_2_ [74], [75].

For cell viability assays neuroblastoma and medulloblastoma cells were seeded in 96-well plates at a density of 3^′^000–10^′^000 cells/well and grown for 48–118 hrs in cell culture containing high (10%) serum. Cell proliferation was analyzed by the CellTiter 96®AQ_ueous_One Solution Cell Proliferation Assay (Promega) according to the manufacturer's instructions. For detection of apoptosis, NB and MB cells were seeded on 6-well plates and incubated for 24 h in the presence or absence of PIK75, R1507 or cisplatin. After lysis of the cells the protein samples were analyzed by SDS-PAGE and Western blot with anti-PARP and anti-caspase-3 antibodies. Additionally, apoptosis was analyzed by caspase 3/7 activation using the Caspase-Glo 3/7 Assay (Promega), according to the manufacturer's instruction.

### Western blotting

Cell lysates were prepared in RIPA buffer (50 mM Tris-Cl, pH 6.8, 100 mM NaCl, 1% Triton X-100, 0.1% SDS) supplemented with Complete Mini Protease Inhibitor Cocktail (Roche Applied Sciences) and with the phosphatase inhibitors β-glycerophosphate (20 mM) and Na_3_VO_4_ (200 mM) and normalized using a bicinchoninic acid (BCA) protein assay (Pierce, Rockford, IL, USA). Cell lysates were separated by sodium dodecyl sulfate-polyacrylamide gel electrophoresis (SDS-PAGE), transferred to a hydrophobic polyvinylidenedifluoride (PVDF) membrane (Hybond-P; Amersham Biosciences, GE Healthcare, UK) and immunoblotted with the indicated antibodies prior to chemiluminescent detection (SuperSignal West Femto Maximum Sensitivity Substrate (Thermo Fisher Scientific Inc., Rockford, IL, USA).

### siRNA transfection

LAN-1 neuroblastoma cells (10^4^ cells per well) were plated in 96-well plates and transfected 24 h later with siRNA targeting the IGF-1R or non-targeting siRNA (Ambion, Applied Biosystems, Foster City, CA, USA) at a final concentration of 30 nM, using Lipofectamine 2000 following the manufacturer's instructions (Invitrogen, Carlsbad, CA, USA). Twenty-four hours following transfection, cells were treated with R1507 (at different concentrations). Cell viability was analyzed after 72 h.

### Statistical analysis

Analysis of variance was used to assess statistical significance of differences between groups. p values <0.05 were considered as significant.

## Results

### Anti-proliferative activity of R1507 and PIK75 in panels of neuroblastoma and medulloblastoma cell lines

We have previously described panels of neuroblastoma and medulloblastoma cell lines, which were characterized for expression of components of the IGF-1R/PI3K signaling pathway [20], [70], [75]. In the present study, the impact of the humanized anti-IGF-1R antibody R1507 was evaluated on cell proliferation *in vitro* in the panels of neuroblastoma and medulloblastoma cell lines (Fig. 1). The antibody displayed anti-proliferative activity in 2 out of 8 neuroblastoma cell lines, namely SH-SY5Y and LAN1 (Fig. 1A and 2A). In SH-SY5Y, R1507 induced a maximal decrease in cell viability of 60% at 12.5 μg/ml (Fig. 1A). In LAN1 a maximal activity of ∼25% reduction in cell proliferation was observed (Fig. 2A). R1507 showed anti-proliferative activity in 2 out of 5 medulloblastoma cell lines, namely PFSK and D458 (Fig. 1B). In D458, a maximal decrease in cell proliferation (60%) was observed at 15 μg/ml, while in PFSK the maximal effect was 40% inhibition of the response (Fig. 1B). In NB and MB cell lines, the activity of R1507 was cell-line specific and the antibody had a profile similar to the IGF-1R tyrosine kinase inhibitor NVP-AEW541 [20]. NVP-AEW541 was more active in SH-SY5Y and LAN-1 than in other neuroblastoma cell lines [20], and PFSK were more sensitive to NVP-AEW541 than DAOY and UW228 cells. The expression levels of the IGF-1R in NB cells did not correlate with the activity of R1507, as previously observed with NVP-AEW541 [20]. Similarly, in the MB cell line panel which was previously analyzed for IGF-1R expression [75], there was apparently no correlation between receptor expression and the activity of R1507.

The impact of the class I_A_ PI3K inhibitor PIK75 was evaluated on cell proliferation *in vitro* in the panels of neuroblastoma and medulloblastoma cell lines (Fig. 3). The inhibitor displayed strong anti-proliferative activity in the neuroblastoma cell line panel, with IC_50_ values in the range of 50–100 nM (Fig. 3A). In medulloblastoma cell lines, PIK75 was more active in D341 and D458 cells (IC_50_ = 20 nM) than in UW228 cells. The activity of PIK75 in DAOY and PFSK cells was previously described [75].

### Impact of R1507 and PIK75 on intracellular signaling pathway activation

The impact of R1507 and PIK75 on the activation status of the Akt/mTOR pathway in NB cell lines was investigated by Western blot analysis (Fig. 4). R1507 strongly affected the activation status of Akt and the phosphorylation of the mTOR downstream target ribosomal S6 protein in the R1507-responsive NB and MB cell lines. (SH-SY5Y in Fig. 4A, LAN1 cells Fig. 4B, as well as in medulloblastoma PFSK cells Fig. 4D). Concentrations of 6.25–100 μg/ml R1507 reduced Akt^Ser473^ phosphorylation, whereas only concentrations of more than 6.25 or 12.5 were needed to reduce S6^Ser235/236^ phosphorylation. In the R1507-insensitive NB cell line WAC2, the antibody did not induce a comparable response in terms of inhibition of the PI3K signaling pathway (Fig. 4C).

Also PIK75 was able to inhibit Akt/mTOR activation, as seen on the decreases in the phosphorylation of S6 protein in both NB cell lines treated (Fig. 4A+B), although only in LAN-1 cells the phosphorylation of Akt^Ser473^ was affected (Fig. 4B). The inhibitory effects of PIK75 in Akt/mTOR signaling in MB cell lines cells were previously described in [75] and correlate with the effects observed in neuroblastoma cells.

### IGF-1R expression and activation in neuroblastoma and medulloblastoma cell lines

Further, we wanted to investigate the impact of R1507 on the expression and activation status on the IGF-1 receptor in MB and NB cell lines. Pre-treatment with R1507 inhibited the phosphorylation but not the expression level of IGF-1R in DAOY and LAN1 cells after IGF-1 stimulation (Fig. 4E+F).

### Combination of R1507 with standard chemotherapeutic agents in neuroblastoma and medulloblastoma cell lines

We have previously shown that the IGF-1R tyrosine kinase inhibitor NVP-AEW541 enhances the effects of cisplatin on cell proliferation and apoptosis in neuroblastoma cell lines [20]. In support of this finding, the concomitant treatment of the R1507-responsive SH-SY5Y neuroblastoma cell line with R1507 and cisplatin resulted in additive effects on cell proliferation (Fig. 5A). For neuroblastoma WAC2 cells that did not respond to R1507 in single treatment (Fig. 1A), there was no additional effect of R1507 in combination with cisplatin, doxorubicin or etoposide (Fig. S1). In medulloblastoma PFSK and UW228 cells, the combination of R1507 and cisplatin was more effective than the single agents (Fig. 6A+B). This is not surprising for PFSK, that also showed sensitivity to R1507 alone, but in UW228, cisplatin seems to confer R1507 sensibility. In R1507-insensitive DAOY cells no such effect was observed (Fig. 6C).

### Combination of PIK75 with standard chemotherapeutic agents in neuroblastoma cells

In the neuroblastoma cell lines LAN1 and WAC2, the concomitant treatment with PIK75 and doxorubicin or etoposide resulted in additive effects on cell proliferation (Fig. 5B+C). At selected, relatively low concentrations, where PIK75, doxorubicin or etoposide alone reduced cell proliferation to 60–80%, combination treatments of PIK75 and one of the chemotherapeutic agents brought reductions to 40–50%. Our previous work on medulloblastoma has shown that PIK75 sensitizes medulloblastoma cell lines to doxorubicin [75].

### Inhibitors of the RTK-PI3K-mTOR signaling in medulloblastoma

Beside classical chemotherapeutic agents used for cancer treatment, targeted therapies involving inhibitors of the RTK-PI3K signaling axis are considered to be a promising approach in cancer treatment. To investigate the role of receptor tyrosine kinases signaling in medulloblastoma, the effect of different targeted therapies was additionally studied. *In vitro*, the IGF-1R inhibitor NVP-AEW541 reduced the cell viability of the MB cell line DAOY with an IC50 of 2.5 µM and a maximal reduction of cell proliferation to 5% (Fig. 7A, 8C), whereas in UW228 cells concentrations higher than 10 μM were needed to provoke a response (Fig. 7B). Also targeting the EGFR with gefinitib or erlotinib was more effective in DAOY than in UW228 cells (Fig. 7A+B), with DAOY responding from concentrations higher than 2.5 μM. In UW228 cells erlotinib did not cause any effect, whereas gefitinib treatment reduced cell viability to 5% at the highest concentration tested (20 μM) (Fig. 7B). Rapamycin, a commonly used mTOR inhibitor, led to 50% reduction in cell proliferation in DAOY cells (0.0625 μg/ml) (Fig. 8D). UW228 cells responded to rapamycin with a maximum decrease in cell viability of 30% (2 μg/ml) (Fig. 7B). Imatinib, an inhibitor of the RTKs PDGFR and c-Kit, was able to reduce the cell proliferation in DAOY cells to 15% (Fig. 7A, 8B) and in UW228 cells to 40% (Fig. 7B). Before, it could be shown that the concomitant treatment of the IGF-1R antibody R1507 with cisplatin resulted in additive effects in R1507-responsive cell lines, or even was able to sensitize the R1507-non-responsive cell line UW228, but not DAOY (Fig. 6). Furthermore it was of interest, whether a concomitant treatment of different RTK/PI3K/mTOR inhibitors and R1507 could cause sensitization effects in non-responsive DAOY cells. Co-targeting EGFR (gefitinib) or PDGFR and c-Kit (imatinib) together with the IGF-1R (R1507) was not able to sensitize DAOY cells, and interestingly, even caused negative effects, meaning the combination treatment was less effective than the single agent (Fig. 8A+B). R1507, in combination with the IGF-1R inhibitor NVP-AEW541 or the mTOR inhibitor rapamycin could not further increase the effect of the single treatment (Fig. 8C+D).

### The role of the IGF-1R/PI3K/Akt signaling axis on cell survival in neuroblastoma and medulloblastoma

The impact of the IGF-1R/PI3K/Akt signaling axis on survival of NB and MB cells was investigated by treating the cells with increasing concentrations of R1507 or PIK75, and apoptosis was measured by PARP cleavage and caspase-3activation, both markers of apoptotic activity. Whereas PIK75 treatment led to enhanced PARP and pro-caspase 3 cleavage in LAN1 and SH-SY5Y cells (Fig. 9A+B) or to an increase in the caspase 3/7 activity in WAC2 NB cells (Fig. 9C), a comparable induction of apoptosis could not be observed with R1507 treatment in NB cells or by treating the MB cell line PFSK (Fig. 9A+B).

### Activity of R1507 and PIK75 in chemoresistant NB cell lines

We next investigated whether R1507 or PIK75 had also anti-proliferative effects in neuroblastoma cell lines with acquired resistance to standard chemotherapeutic agents (LAN1R). R1507 displayed no significant anti-proliferative activity in LAN1 cells with acquired resistance to doxorubicin (Fig. 2A). In contrast, PIK75 displayed almost comparable anti-proliferative activity in either parental LAN1 or their chemoresistant counterparts (Fig. 2B). Western blot analysis of the protein expression of LAN1R cells showed that these cells express reduced levels of IGF-1R and p110α compared to the parental cell line LAN1. In addition, the phosphorylation levels of ERK1/2 and AKT at the positions Ser^473^ and Thr^308^ were also lower in LAN1R than in LAN1 (FIG. 2C). In order to investigate whether down-regulation of the IGF-1R could reduce the sensitivity of NB cells to R1507, observation, LAN1 cells were transiently transfected with siRNA targeting the receptor. At 96 h after transfection, a 50% reduction of IGF-1R expression was still observed (Fig. 2D). LAN1 cells transfected with IGF-1R siRNA displayed reduced proliferation, when compared to control cells transfected with non-targeting siRNA (Fig. 2D). In addition, IGF-1R silencing completely abrogated the response to R1507 (Fig. 2D), confirming that down-regulation of the receptor in the LAN1R cells contributes to the lack of effect of R1507 in these cells (Fig. 2A).

## Discussion

In the present report we have evaluated the anti-proliferative activity of the humanized anti-IGF-1R antibody R1507 in the embryonal tumors neuroblastoma and medulloblastoma *in vitro*. As a single agent, R1507 was effective in a subset of neuro- and medulloblastoma cell lines, while a majority of cell lines did not respond. The profile of R1507 in neuro- and medulloblastoma was similar to the IGF-1R tyrosine kinase inhibitor NVP-AEW541 in terms of the identity of the cell lines which were sensitive to the single agent [20]. The expression levels of the IGF-1R in MB and NB cells did not correlate with the activity of R1507, as previously observed with NVP-AEW541 [20]. The constitutive activation of downstream signaling pathways such as Akt and mTOR/S6K may modulate the sensitivity of the cells to R1507, as previously reported for NVP-AEW541 [20]. In neuroblastoma cell lines that were sensitive to R1507 as single agent, the effects of R1507 and chemotherapy (cisplatin, doxorubicin and etoposide) were additive, a result which was also observed with NVP-AEW541 [20]. However, neuroblastoma cells which were not sensitive to R1507, showed also no additive effects in cell growth inhibition to when combined with chemotherapies. By contrast, in medulloblastoma R1507 showed strong additive effects with cisplatin not only in MB cells which were initially sensitive to R1570 (PFSK), but also MB cells which were insensitive to R1507 as a single agent (UW228). Analysis of the mechanisms of action revealed that R1507 inhibits cell growth by attenuation of the AKT/mTOR signaling pathway in neuroblastoma and medulloblastoma cells. Similar observations were obtained by inhibition of IGF-1R with NVP-AEW541 [20]. Interestingly, the concomitant treatment with R1507 and inhibitors of the RTK/PI3K/mTOR signaling could not overcome the resistance of insensitive DAOY cells, resulted in combination with gefitinib and imatinib even in negative effects, compared to when they were used as single agent. Generally, DAOY cells responded to single treatments targeting the RTK/PI3K/mTOR signaling, such as IGF-1R (NVP-AEW541), EGFR (gefinitib, erlotinib), PDGFR and c-Kit (imatinib), and mTOR (rapamycin). Cell growth of the cell line UW228 was mostly not affected or higher concentrations were needed to induce a response by use of the same agents.

Our previous work using RNAi targeting of classI_A_ PI3K isoforms has revealed that targeting these enzymes in neuroblastoma and medulloblastoma cell lines can induce apoptosis and decrease cell proliferation [70], [75]. These results are supported by the findings presented here, which show that PIK75 displays a broad anti-proliferative activity in neuroblastoma cell lines. Also in medulloblastoma, we observed that PIK75 has anti-proliferative activity, but one cell line (UW228) was rather resistant to the drug. The exact mechanism(s) underlying this observation are at present unclear, but may be caused by an enhanced activation of Erk1/2. A decrease in activity of class I_A_ PI3K inhibitors has been observed previously in cell lines with mutant *KRAS* and attributed to the enhanced activation of the Erk pathway [76]. The combination of PIK75 with chemotherapy (doxorubicin and etoposide) showed enhanced cell growth inhibition as compared with single agent treatment in neuroblastoma cell lines. Consistent with these findings, a recent report demonstrated that PI103 a dual inhibitor against p110α and mTOR strongly synergizes with various chemotherapeutics including doxorubicin, etoposide and cisplatin [77]. In medulloblastoma, our previous work has also demonstrated the anti-proliferative effects for PIK75 in combination with different chemotherapeutic agents [75].

Because neuroblastoma and medulloblastoma cells may express a variety of different growth factor receptors, we and others have postulated that targeting individual receptors may not always provide the best therapeutic option [20], [70], [75]. To overcome this problem, an alternative approach was proposed, which is based on targeting downstream signaling molecules that are regulated by different growth factor receptors to transmit the proliferative message. Our findings support this approach, since we observed that generally a bigger number of NB and MB cell lines most likely responded to PIK75 than to R1507. Importantly, PIK75 effectively inhibited proliferation in a chemoresistant neuroblastoma cell line in a comparable manner as in the parental cell line, demonstrating its broad anti-proliferative activity. By contrast, R1507 was ineffective in the chemoresistant neuroblastoma cells, which was most likely caused by reduced expression of the IGF-1R. The activation status of the AKT/mTOR pathway was also found to be reduced in the chemoresistant cells, pointing that this signaling pathway may not be responsible for the acquired chemoresistant phenotype of the cells. However, our previous findings in medulloblastoma cells showed elevated levels of phosphorylated Akt as a consequence of short time exposure with doxorubicin [75]. The molecular mechanisms underlying these observations are at present unclear, but may be of importance, in view of the fact that some clinical trials have been initiated with R1507 in patients previously treated with chemotherapy [78].

## Supporting Information

Figure S1 **The NB cell line WAC2 is resistant to the combinatorial treatment of R1507 with chemotherapeutic agents.** Neuroblastoma WAC2 cells treated with R1507 in combination with chemotherapy, incubated for 48 hours. Already shown to be insensitive to R1507 alone (Fig. 1A), WAC2 cells are here shown also to be insensitive to R1507 in combination with chemotherapeutic agents (A) doxorubicin and (B) cisplatin. Error bars represent ±S.D. of means from 1 to 2 experiments, each with 8 replicates. For combination experiments with R1507-sensitive neuroblastoma cell lines, see Fig. 5.
(TIF)
