# Supplementary figures and images for: Novel Agents Targeting the IGF-1R/PI3K Pathway Impair Cell Proliferation and Survival in Subsets of Medulloblastoma and Neuroblastoma
Source: PLoS One. 2012 Oct 8;7(10):e47109. doi: 10.1371/journal.pone.0047109 (PMC3466180; doi:10.1371/journal.pone.0047109)

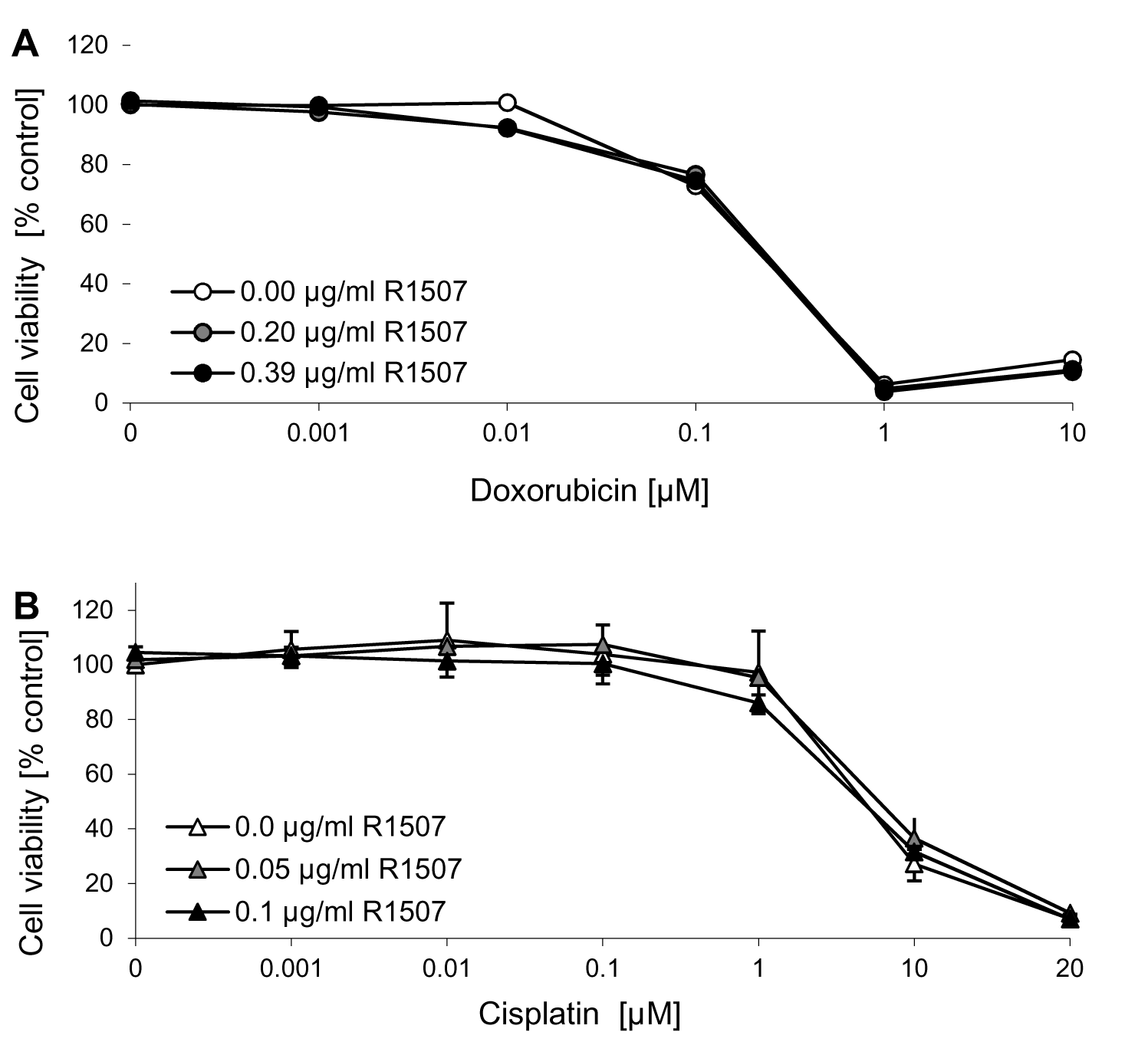

Supplement: Figure S1 — The NB cell line WAC2 is resistant to the combinatorial treatment of R1507 with chemotherapeutic agents. Neuroblastoma WAC2 cells treated with R1507 in combination with chemotherapy, incubated for 48 hours. Already shown to be insensitive to R1507 alone (Fig. 1A), WAC2 cells are here shown also to be insensitive to R1507 in combination with chemotherapeutic agents (A) doxorubicin and (B) cisplatin. Error bars represent ±S.D. of means from 1 to 2 experiments, each with 8 replicates. For combination experiments with R1507-sensitive neuroblastoma cell lines, see Fig. 5. (TIF) [file pone.0047109.s001.tif]
